# Locations and Reasons for Initial Testing for Hepatitis C Infection — Chronic Hepatitis Cohort Study, United States, 2006–2010

**Published:** 2013-08-16

**Authors:** Joseph A. Boscarino, Stuart C. Gordon, Loralee B. Rupp, Mark A. Schmidt, Vinutha Vijayadeva, Anne Moorman, Fujie Xu, Scott D. Holmberg, Stephen C. Ko

**Affiliations:** Geisinger Health System, Danville, Pennsylvania; Henry Ford Health System, Detroit, Michigan; Permanente Northwest, Portland, Oregon; Kaiser Permanente Hawaii, Honolulu, Hawaii; Div of Viral Hepatitis, National Center for HIV/AIDS, Viral Hepatitis, STD, and TB Prevention; EIS Officer, CDC

Chronic hepatitis C virus (HCV) infection causes substantial morbidity and mortality in the United States (1). Testing and treatment of asymptomatic persons might avert progression to more advanced disease. In 1998, CDC published guidelines for HCV testing based on risk factors for infection; however, recent studies indicate that at least one half of all persons living with HCV infection in the United States are unaware of their infection status (2–4). To increase testing rates, in 2012 CDC recommended one-time testing of all persons born during 1945–1965 (5). To better understand where and why persons with chronic HCV infection sought their initial testing, 2006–2010 data were analyzed from a survey conducted as part of the ongoing Chronic Hepatitis Cohort Study (6). Of 4,689 patients with HCV infection who responded to the survey, 60.4% reported that their initial HCV test occurred in a physician’s office. CDC’s risk-based indications (e.g., injection drug use and hemodialysis) were cited by 1,045 (22.3%) of the patients as reasons for testing, whereas clinical indications (e.g., abnormal liver function tests or liver-related symptoms such as jaundice) were cited by 2,121 (45.2%), suggesting that many HCV infections were identified only after the patient had become symptomatic. Promoting U. S. Preventive Services Task Force (7) and CDC recommendations for testing (5) and identifying strategies that help physicians implement HCV testing in their offices might help facilitate timely identification of HCV infection and reduce morbidity and mortality.

The Chronic Hepatitis Cohort Study follows patients with confirmed chronic HCV or hepatitis B virus infection who receive care at four integrated health-care systems in the United States (3,6): Geisinger Health System, Danville, Pennsylvania; Henry Ford Health System, Detroit, Michigan; Kaiser Permanente Hawaii, Honolulu, Hawaii; and Northwest Permanente, Portland, Oregon. Of 12,529 patients aged ≥18 years who met the inclusion criteria for confirmed chronic HCV infection (6), 10,380 (82.8%) were sampled randomly for the current analysis. After excluding 1,451 patients who died and 828 who could not be contacted because of an invalid telephone number or address, incarceration, long-term care, or because of a physician’s request that contact should not be made, the remaining 8,101 (64.7%) patients were surveyed by U.S. mail or telephone during 2011–2012. Up to eight telephone contact attempts were made; a small incentive was offered to encourage participation. The study protocol was reviewed and approved by an institutional review board approved by the federal Office for Human Research Protections at each participating site.

The survey was designed to collect data regarding the location and reasons for initial HCV testing. Participants were asked to choose from a list of reasons for HCV testing. Their responses were then grouped and analyzed in four categories: 1) CDC risk indications, according to the 1998 guidelines for testing (e.g., injection drug use and hemodialysis); 2) clinical indications (e.g., abnormal liver function tests or liver-related symptoms such as jaundice or abdominal pain); 3) institutional requirements (e.g., from blood banks, insurance or health maintenance organizations, prison, work/school, or the military); and 4) other miscellaneous reasons, including a doctor recommendation, “thought I was exposed,” spouse’s recommendation, foreign-born (from a country where hepatitis is endemic), and sexual contact with an HCV-infected person. Reasons for testing were not mutually exclusive; patients could choose more than one reason.

Of the 8,101 patients contacted, 4,689 (57.9%) completed the survey. Compared with nonrespondents, survey participants were slightly older (mean age: 57.4 years compared with 56.9 years, p=0.003), more likely to be white (72.8% compared with 61.4%, p<0.001), and more likely to be women (43.9% compared with 38.0%, p<0.001).

Of the 4,689 participants, 3,663 (78.1%) were born during 1945–1965; 87.4% had a high school diploma or its equivalent; 98.1% had insurance; 45.5% were employed; and 23.2% received disability payments (Table 1). Most respondents (60.4%) reported receiving the HCV test in a physician’s office (Table 2). For those born during 1945–1965: 62.1% were tested in physicians’ offices, 9.4% in blood banks or at blood drives, 7.4% in public health or specialty clinics, and 5.4% in inpatient settings (Table 2). For those born before 1945 or after 1965, a smaller proportion (54.3%) of tests occurred in physicians’ offices, whereas testing in clinics (11.9%) and inpatient settings (7.5%) constituted larger proportions.

The 4,689 participants reported 7,649 reasons for their initial HCV test. Of the total, 3,473 responses (45.4%) were “miscellaneous reasons” not included in CDC’s risk indications for testing (Table 3).

Among the 4,689 survey participants, clinical indications were reported by 2,121 (45.2%) as a reason for testing and CDC risk indications by 1,045 (22.3%). Among the 1,045 participants citing CDC risk indications, 986 (94.4%) reported injection drug use. Institutional requirements were reported by 781 (16.7%), and doctor-recommended testing was reported by 1,725 (36.8%) participants (Table 3).

For the 3,663 participants born during 1945–1965, clinical indications were cited by 1,713 (46.8%) participants, with 781 (21.3%) reporting CDC risk indications as a reason for their initial HCV test. Among those born during 1945–1965, institutional requirements were reported as a reason by 638 (17.4%), and 1,319 (36.0%) reported doctor recommendations as a reason for testing (Table 3).

What is already known on this topic?

Since 1998, CDC has recommended testing for viral hepatitis C virus (HCV) infection among persons most likely to be infected. These recommendations have led to significant progress in identifying patients with HCV infection. However, a substantial percentage of patients with HCV infections have not been tested and remain unaware of their infection.

What is added by this report?

An analysis of 2006–2010 data from the Chronic Hepatitis Cohort Study indicated that a substantial proportion of HCV-infected patients were tested only after clinical indications that their infection had progressed and became symptomatic. Of the 4,689 patients with HCV infection who responded to the survey, 45.2% reported clinical indications as a reason for testing, with 78.1% born during 1945–1965, the birth cohort recommended by CDC for one-time HCV testing.

What are the implications for public health practice?

Promoting CDC’s risk factor and birth cohort–based recommendations for HCV testing, along with implementing HCV testing in physicians’ offices and other venues can allow timely identification of HCV infections and reduce HCV-related morbidity and mortality.

## Editorial Note

The Chronic Hepatitis Cohort Study survey data analyzed in this report indicate that most initial HCV tests occurred in a physician’s office, and nearly half of those infected with HCV only sought testing after experiencing clinical indications of liver disease. Testing for HCV infection in a location other than a physician’s office occurred for about one third of respondents. Other locations included clinics, inpatient settings, and emergency departments. Other studies have shown a greater proportion (50.7%) of testing in locations other than a physician’s office or laboratory (8). The results in this report suggest that, in addition to increasing testing in physicians’ offices, other locations might be important for increasing the number of HCV-infected persons who are tested and referred to care.

Less than one fourth of HCV-infected patients gave CDC risk indications as a reason for testing, but many reported various other reasons (e.g., doctor recommendation, “thought I was exposed,” and having many sex partners) that were not included in the 1998 CDC recommendations (2). Other reasons for testing (e.g., multiple sex partners) also have been reported (8,9). Responses in the study, such as “thought I was exposed” or doctor recommendation, suggest improved patient education could enhance patient’s understanding of the risks for HCV infection.

This analysis indicates that approximately four out of five patients in this study of 2006–2010 data were born during 1945–1965, and therefore were within the birth cohort targeted in the 2012 CDC HCV testing guidelines (5). Only 21.3% of those born during 1945–1965 gave a reason for testing (injection drug use or hemodialysis) that was included in the earlier 1998 CDC risk indications.

CDC is identifying strategies to help health-care providers implement its new HCV testing guidelines, which target all persons born during 1945–1965. These strategies include simplification of HCV testing algorithms in primary care and public health settings, development of national educational strategies for testing those born during 1945–1965, and supporting evidence-based care models that enhance delivery of high-quality HCV assessment and management (10).

The findings in this report are subject to at least three limitations. First, patients surveyed from the four health sites were not from a nationally representative sample, so these results are not generalizable to the U.S. population of persons with HCV infection. Almost all patients were covered by some form of health insurance, and risk-based behaviors (e.g., injection drug use) were less common in this group than has been observed in surveillance-based studies (9). Second, only 57.9% of persons contacted completed the survey, which might have resulted in response bias. Finally, the long interval between initial testing and time of interview and the potential for inconsistency between self-reported reasons for testing and a health-care provider’s rationale for testing might have resulted in recall bias.

This survey of patients with HCV infection enrolled in the Chronic Hepatitis Cohort Study indicates that nearly four out of five participants were born during 1945–1965, a cohort for whom CDC recommends HCV testing in its 2012 guidelines (5). Because a substantial proportion of HCV infections were identified after testing for clinical indications and few patients reported the 1998 CDC risk indications as a reason for initial testing, these data further support the CDC recommendation for testing all persons in the birth cohort of 1945–1965 in addition to risk-based testing. Physicians’ offices and other locations might be important venues for implementing these guidelines to increase HCV testing.

## Figures and Tables

**TABLE 1:** Characteristics of HCV-infected patients (N = 4,689) — Chronic Hepatitis Cohort Study, United States, 2006–2010

Characteristic	No.	(%)*
**Birth year**		
After 1965	587	(12.5)
1945–1965	3,663	(78.1)
Before 1945	439	(9.4)
**Sex**		
Men	2,628	(56.1)
Women	2,061	(43.9)
**Race**		
White	3,328	(72.8)
Black or African American	888	(19.4)
Asian	143	(3.1)
American Indian or Alaska Native	138	(3.0)
Native Hawaiian or Other Pacific Islander	76	(1.7)
Unknown	116	—
**Hispanic ethnicity**		
Yes	208	(4.6)
No	4,317	(95.4)
Unknown	164	—
**Education**		
Less than high school diploma	529	(12.6)
High school/General Equivalency Diploma	1,192	(28.5)
Some college/Technical school	1,507	(36.0)
College graduate or higher	961	(22.9)
Unknown	500	—
**Health-care coverage**		
Private	2,941	(66.0)
Medicare plus	812	(18.2)
Medicaid	459	(10.3)
Medicare only	161	(3.6)
None	86	(1.9)
**Employment**		
Part-time/Full-time	2,035	(45.5)
Disability	1,090	(23.2)
Retired	897	(20.1)
Unemployed	448	(9.6)
Unknown	219	—

**Abbreviation:** HCV = hepatitis C virus.

* Missing values were excluded from percentage distributions.

**TABLE 2:** Locations for testing of HCV-infected patients — Chronic Hepatitis Cohort Study, United States, 2006–2010

	Year of birth							
	
	Total (N = 4,689)		Before 1945 (n = 439)		1945–1965 (n = 3,663)		After 1965 (n = 587)	
				
Location	No.	(%)	No.	(%)	No.	(%)	No.	(%)
Physician office	**2,832**	**(60.4)**	276	(62.9)	2,275	(62.1)	281	(47.9)
Blood bank or blood drive	**424**	**(9.0)**	31	(7.0)	345	(9.4)	48	(8.2)
Clinic*	**393**	**(8.4)**	24	(5.5)	271	(7.4)	98	(16.7)
Hospital inpatient†	**275**	**(5.9)**	26	(5.9)	198	(5.4)	51	(8.7)
Insurance exam site	**141**	**(3.0)**	8	(1.8)	122	(3.3)	11	(1.9)
Emergency department	**141**	**(3.0)**	10	(2.3)	100	(2.7)	31	(5.3)
Prison	**71**	**(1.5)**	2	(0.5)	46	(1.3)	23	(3.9)
Army	**20**	**(0.4)**	2	(0.5)	18	(0.5)	0	(0)
Other	**99**	**(2.1)**	8	(1.8)	74	(2.0)	17	(2.9)
Unknown	**293**	**(6.3)**	52	(11.8)	214	(5.8)	27	(4.6)

**Abbreviation:** HCV = hepatitis C virus.

* Clinics included prenatal/family planning, sexually transmitted disease, infectious disease, tuberculosis, drug treatment, community, school/work, and unspecified clinics.

† Included obstetrics wards.

**TABLE 3:** Reported reasons for testing* among HCV-infected patients (N = 4,689), by year of birth — Chronic Hepatitis Cohort Study, United States, 2006–2010

	Year of birth							
	
	Total reasons (N = 7,649)		Before 1945 (n = 645)		1945–1965 (n = 5,926)		After 1965 (n = 1,078)	
				
Category of reasons	No.	(%)	No.	(%)	No.	(%)	No.	(%)
**CDC risk indications**	**1,045**	**(13.7)**	39	(6.0)	781	(13.2)	225	(20.9)
Injection drug use	**986**	**(94.4)**	31	(79.5)	736	(94.2)	219	(97.3)
Hemodialysis	**59**	**(5.6)**	8	(20.5)	45	(5.8)	6	(2.7)
**Clinical indications**	**2,121**	**(27.7)**	219	(34.0)	1,713	(28.9)	189	(17.5)
Abnormal liver function test	**1,497**	**(70.6)**	158	(72.1)	1,212	(70.8)	127	(67.2)
Liver symptoms†	**624**	**(29.4)**	61	(27.9)	501	(29.2)	62	(32.8)
**Institutional requirement**	**781**	**(10.2)**	57	(8.8)	638	(10.8)	86	(8.0)
Blood donor	**506**	**(64.8)**	38	(66.7)	410	(64.3)	58	(67.4)
Insurance/HMO	**145**	**(18.6)**	9	(15.8)	126	(19.7)	10	(11.6)
Prison	**80**	**(10.2)**	6	(10.5)	57	(8.9)	17	(19.8)
Work/School	**39**	**(5.0)**	2	(3.5)	36	(5.6)	1	(1.2)
Military	**11**	**(1.4)**	2	(3.5)	9	(1.4)	0	—
**Miscellaneous**	**3,473**	**(45.4)**	294	(45.6)	2,618	(44.2)	561	(52.0)
Doctor recommendation	**1,725**	**(49.7)**	205	(69.7)	1,319	(50.4)	201	(35.8)
“Thought I was exposed”	**639**	**(18.4)**	28	(9.5)	458	(17.5)	153	(27.3)
Sexual contact with HCV	**338**	**(9.7)**	16	(5.4)	243	(9.3)	79	(14.1)
Many sex partners	**228**	**(6.6)**	8	(2.7)	177	(6.8)	43	(7.7)
Household contact with HCV	**200**	**(5.8)**	10	(3.4)	154	(5.9)	36	(6.4)
Spouse recommendation	**76**	**(2.2)**	8	(2.7)	58	(2.2)	10	(1.8)
MSM	**46**	**(1.3)**	0	(0)	31	(1.2)	15	(2.7)
Born in country with endemic HCV	**32**	**(0.9)**	6	(2.0)	22	(0.8)	4	(0.7)
Other	**189**	**(5.4)**	13	(4.4)	156	(6.0)	20	(3.6)
**Unknown**	**229**	**(3.0)**	36	(5.6)	176	(3.0)	17	(1.6)

**Abbreviations:** HCV = hepatitis C virus; MSM = men who have sex with men; HMO = health maintenance organization.

* Categories were not mutually exclusive; more than one response was allowed per patient.

† Liver-related symptoms included but were not limited to 1) jaundice/yellowing of the eyes and skin and 2) abdominal pain.

## References

[1] Institute of Medicine (2010). Hepatitis and liver cancer: a national strategy for prevention and control of hepatitis B and C.

[2] CDC (1998). Recommendations for prevention and control of hepatitis C virus (HCV) infection and HCV-related chronic disease. MMWR.

[3] Spradling PR, Rupp L, Moorman AC (2012). Hepatitis B and C virus infection among 1.2 million persons with access to care: factors associated with testing and infection prevalence. Clin Infect Dis.

[4] Denniston MM, Klevens RM, McQuillan GM, Jiles RB (2012). Awareness of infection, knowledge of hepatitis C, and medical follow-up among individuals testing positive for hepatitis C: National Health and Nutrition Examination Survey 2001–2008. Hepatology.

[5] CDC (2012). Recommendations for the identification of chronic hepatitis C virus infection among persons born during 1945–1965. MMWR.

[6] Moorman AC, Gordon SC, Rupp LB (2013). Baseline characteristics and mortality among people in care for chronic viral hepatitis: the Chronic Hepatitis Cohort Study. Clin Infect Dis.

[7] Moyer VA (2013). Screening for hepatitis C virus infection in adults: US Preventive Services Task Force recommendation statement. Ann Intern Med.

[8] Tohme RA, Xing J, Liao Y, Holmberg SD (2013). Hepatitis C testing, infection, and linkage to care among racial and ethnic minorities in the United States, 2009–2010. Am J Public Health.

[9] Mahajan R, Liu SJ, Klevens RM, Holmberg SD (2013). Indications for testing among reported cases of HCV infection from enhanced hepatitis surveillance sites in the United States, 2004–2010. Am J Public Health.

[10] CDC (2013). Testing for HCV infection: an update of guidance for clinicians and laboratorians. MMWR.

